# TikTok as a Health Information Source: Assessment of the Quality of Information in Diabetes-Related Videos

**DOI:** 10.2196/30409

**Published:** 2021-09-01

**Authors:** Wenwen Kong, Shijie Song, Yuxiang Chris Zhao, Qinghua Zhu, Ling Sha

**Affiliations:** 1 Nanjing Hospital of Chinese Medicine Affiliated to Nanjing University of Chinese Medicine Nanjing China; 2 School of Information Management Nanjing University Nanjing China; 3 School of Economics and Management Nanjing University of Science and Technology Nanjing China; 4 Nanjing Jiangning Maternal and Child Health and Family Planning Service Center Nanjing China

**Keywords:** diabetes, information quality, infodemiology, social media, short video apps, TikTok

## Abstract

**Background:**

Diabetes has become one of the most prevalent chronic diseases, and many people living with diabetes use social media to seek health information. Recently, an emerging social media app, TikTok, has received much interest owing to its popularity among general health consumers. We notice that there are many videos about diabetes on TikTok. However, it remains unclear whether the information in these videos is of satisfactory quality.

**Objective:**

This study aimed to assess the quality of the information in diabetes-related videos on TikTok.

**Methods:**

We collected a sample of 199 diabetes-related videos in Chinese. The basic information presented in the videos was coded and analyzed. First, we identified the source of each video. Next, 2 independent raters assessed each video in terms of the completeness of six types of content (the definition of the disease, symptoms, risk factors, evaluation, management, and outcomes). Then, the 2 raters independently assessed the quality of information in the videos, using the DISCERN instrument.

**Results:**

In regard to the sources of the videos, we found 6 distinct types of uploaders; these included 3 kinds of individual users (ie, health professionals, general users, and science communicators) and 3 types of organizational users (ie, news agencies, nonprofit organizations, and for-profit organizations). Regarding content, our results show that the videos were primarily about diabetes management and contained limited information on the definition of the disease, symptoms, risk factors, evaluation, and outcomes. The overall quality of the videos was acceptable, on average, although the quality of the information varied, depending on the sources. The videos created by nonprofit organizations had the highest information quality, while the videos contributed by for-profit organizations had the lowest information quality.

**Conclusions:**

Although the overall quality of the information in the diabetes videos on TikTok is acceptable, TikTok might not fully meet the health information needs of patients with diabetes, and they should exercise caution when using TikTok as a source of diabetes-related information.

## Introduction

Diabetes has become one of the most prevalent chronic diseases throughout the world. According to a recent report by the International Diabetes Federation, the estimated global prevalence of diabetes in people aged 20-79 years reached 493 million in 2019, accounting for 9.3% of the total world population [1]. Crude estimates of diabetes prevalence are 13.0% for US adults [2] and 12.8% for Chinese adults [3]. This high prevalence of diabetes results in huge financial burdens and losses for societies. In 2019, diabetes and related complications led to approximately 4.2 million deaths globally and resulted in US $760 billion of health expenditures [1]. Therefore, there is a pressing need to take action in managing diabetes.

Individuals living with diabetes can actively manage their chronic condition. Early studies suggest that intensive blood glucose control can greatly reduce the risk of complications from the disease [4-6]. However, effective blood glucose control is not an easy task among people living with diabetes. Adequate glycemic controls require a constellation of actions, such as a customized diet, exercise plans, regular self-assessments of blood glucose levels, and optimized medication [7]. According to one study, in 2013, only 25.8% of patients with diabetes had received treatment in China, and only 39.7% of those treated had adequate blood glucose control [8]; such low treatment and adherence rates may be associated with people’s limited knowledge of the disease [9]. People living with diabetes often have diverse needs for information regarding their chronic condition, such as basic information on diabetes and the effectiveness of treatment options, on the sequelae of diabetes, blood glucose control, etc [10]. Nevertheless, they usually encounter many difficulties finding relevant and easy-to-understand information on their conditions [11].

Emerging internet technologies provide opportunities for better health communication and patient education. The internet has shifted the role of patients from passive information recipients to active information seekers [12]. General health information consumers use social media platforms (eg, discussion forums, microblogs, and group chatting) to seek both instrumental advice and emotional support [13-15]. Patients with diabetes who actively use social media for information, according to recent evidence, are associated with having lower glycated hemoglobin values [16]. A possible explanation for this is that social media provides patients with many opportunities to gain health knowledge, thereby increasing patient activation (ie, the ability and willingness that equip patients to take active action in managing their health care) [17]. Therefore, it is essential to utilize social media for better health communication for managing diabetes conditions.

Despite the considerable benefits of social media, its use for health communication has some limitations. In the literature, the quality of the information is the most extensively mentioned concern [18]. The possibility of encountering faulty health information on social media increases risks for patients, who may make health decisions on the basis of inaccurate information [19]. The quality of unmoderated information poses challenges for both patients and health care providers. On one hand, patients need to be able to distinguish high-quality information sources from low-quality ones [20,21]; one the other, health professionals and institutions are expected to respond to and combat health misinformation to protect the public [22]. Therefore, it is important to examine the quality of health information on social media.

Recently, an emerging short video app, TikTok, has attracted the interest of health care researchers [23]. During the COVID-19 pandemic, health-related videos on TikTok were widely viewed and shared. For example, COVID-19–related videos on the app have been watched approximately 93.1 billion times [24]. TikTok contains many videos about diabetes; however, their quality remains unstudied. To address this gap, this study aimed to systematically assess the quality of the information in diabetes-related videos on TikTok.

## Methods

### Search Strategy and Data Extraction

**Figure 1:**
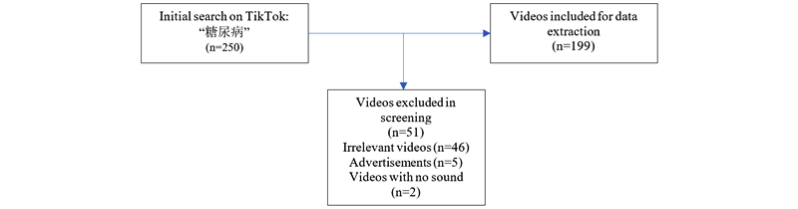
Video screening procedure.

Using the keyword “糖尿病” (“diabetes” in Chinese), we searched TikTok during the period from January 20-25, 2021, and we retrieved the first 250 videos delivered by TikTok’s recommended sorting process. We included the videos directly related to diabetes and excluded videos on other topics, commercial advertisements, and videos with no sound. After the screening, we obtained 199 videos for further data extraction and analysis (Figure 1).

We retrieved and extracted the basic information for each video, including the URL, publication date, name of the uploader, type of uploader (individual vs organization), uploaders’ verification status, length of the video, number of times it was shared, and number of “likes” and comments it received. The extracted data were recorded in Excel (Microsoft Inc).

### Measures

We measured 2 aspects of diabetes-related videos on TikTok: their content and the quality of their information. First, we adopted the coding schema proposed by Goobie et al [25] to rate the quality of six types of content: the definition of the disease, signs/symptoms, risk factors, evaluation, management, and outcomes. Two raters assessed each video independently and scored how sufficiently the video addressed each of the content types on a 3-item scale: 0 points (no content), 1 point (some content), 2 points (extensive content).

To rate the quality of the information, we adopted the DISCERN instrument. According to a systematic review [26], since its publication in 1998, DISCERN has been one of the most widely adopted instruments for assessing the quality of health information. It is a brief questionnaire that enables its users to assess the quality of health information concerning treatment choices [27]. The instrument consists of 16 questions, with response choices based on a 5-point scale, ranging from 1=poor to 5=good. These 16 questions are divided into 3 sections. The first 8 concern the reliability of the publication, such as whether its aims are clear and whether it is relevant, balanced, and unbiased. The scores for this section indicate whether the publication can be trusted as a source of information for choosing a treatment for a particular disease. The 7 questions in the second section focus on the details of treatment choices, such as whether the publication describes how each treatment works and explains its risks and benefits. The scores for this section reflect the quality of the publication’s information about treatment choices (including self-care). The third section consists of 1 final question, based on all the previous ones; it asks users to rate the overall quality of the publication as a source of information about treatment choices. Of note is the fact that, although the original DISCERN instrument was designed for evaluating written publications, it has been widely used for assessing health-related videos. For example, researchers have used it to evaluate YouTube videos informing patients about treatments for cancer [28,29] and diabetes [30].

### Rating process

Two authors (WK and LS) worked on the rating tasks; both are certified physicians who work at endocrinology departments at 2 local hospitals. The raters independently scored each video for its coverage of the 6 types of content and applied the 16 questions of the DISCERN instrument. Interrater reliability was assessed with SPSS (version 22, IBM Corp). The interrater reliability for each of the 6 items relating to video content ranged from 0.813 to 0.981, and all of the reliability coefficients are highly significant at an error margin of 0.1%. The interrater reliability for each of the 16 items of the DISCERN instrument ranges from 0.898 to 0.991, and all of the reliability coefficients are highly significant at an error margin of 0.1%. These results indicate satisfactory interrater reliability.

## Results

### Video Sources

We identified 2 primary sources of the videos: individual and organizational users. We further identified 3 types of video creators among individual users: health professionals, science communicators, and general users. Among organizational users, we identified three types of sources: news agencies, nonprofit organizations, and for-profit organizations.

The results suggest that individual users published most of the videos (n=156, 78.4%). Among individual users, health professionals contributed the most videos (n=138, 69.3%), followed by general users (n=12, 6.0%), and science communicators (n=6, 3.0%). We noted that only 43 videos were uploaded by organizational users, and these accounted for 21.6% of the videos in our sample. Among organizational users, news agencies contributed the most videos (n=31, 15.6%), followed by nonprofit organizations (n=7, 3.5%) and for-profit organizations (n=5, 2.5%) (Table 1).

In our sample, the shortest video lasts only 13 seconds, while the longest lasts 407 seconds. On average, the videos are approximately 1 minute long. All videos were published after 2019. The earliest video had been on TikTok for 589 days, while the latest one was published 3 days prior to the day we collected the data. The videos in the sample received 2.75 million “likes” and 157,700 comments and were shared 305,200 times. Table 2 shows the characteristics of the videos, described by the median numbers across different sources.

**Table 1:** Descriptions of video sources.

Source type		Source description	Videos, n (%)
**Individual users (n=156)**			
	Health professionals	Individuals who identify themselves as health professionals (eg, doctors and nurses)	138 (69.3)
	General users	General users (eg, general health consumers)	12 (6.0)
	Science communicators	Individuals who are engaged in scientific communication (eg, popular science writers)	6 (3.0)
**Organizational users (n=43)**			
	News agencies	News agencies and the press	31 (15.6)
	Nonprofit organizations	Organizations operated for collective, public, or social benefit and public hospitals	7 (3.5)
	For-profit organizations	Organizations that pursue commercial interests	5 (2.5)

**Table 2:** Characteristics of the videos across sources (median numbers).

Source type	Length of video (seconds), median	Days on TikTok, median	“Likes,” median	Comments, median	Times shared, median
Health professionals (n=138)	50.5	131	4191	164	802.5
General users (n=12)	135.5	161	3089.5	78.5	891
Science communicators (n=6)	43	135	24,000	593	4966
News agencies (n=31)	43	90	9917	167	3505
Nonprofit organizations (n=7)	52	200	23,000	194	3023
For-profit organizations (n=5)	63	317	33,000	405	6759

### Video Content

It was not our intention to exclude any type of diabetes during searching and screening. However, we found that most of the videos were about type 2 diabetes mellitus (n=193, 97%). We identified only 4 (2%) videos about gestational diabetes mellitus and 2 (1%) videos about type 1 diabetes mellitus.

**Figure 2:**
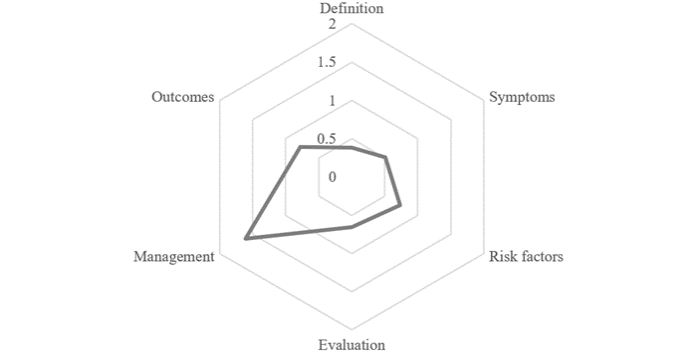
Completeness of video content.

Moreover, we averaged the scores of the 2 raters for each aspect of the video content and obtained scores that ranged over the full 5-point scale, from “no content” to “extensive content.” The results show that more than half of the videos contain little or no content on the definition of the disease, symptoms, or evaluations of diabetes. Overall, 46.2% of the videos contain little or no content on diabetes-related risk, and 66.8% have some or more information on outcomes. Management of diabetes was the most frequent topic in the sample. Overall, 67.8% of the videos sufficiently introduced diabetes management (Table 3). The overall scores for all the videos are given in Figure 2.

**Table 3:** Completeness of video content.

Content	Definition, n (%)	Symptoms, n (%)	Risk factors, n (%)	Evaluation, n (%)	Management, n (%)	Outcomes, n (%)
No content (0 points)	113 (56.8)	115 (57.8)	79 (39.7)	100 (50.3)	14 (7.0)	54 (27.1)
Little content (0.5 points)	27 (13.6)	9 (4.5)	13 (6.5)	8 (4.0)	3 (1.5)	12 (6.0)
Some content (1 point)	52 (26.1)	47 (23.6)	66 (33.2)	54 (27.1)	44 (22.1)	115 (57.8)
Most content (1.5 points)	7 (3.5)	14 (7.0)	17 (8.5)	4 (2.0)	3 (1.5)	5 (2.5)
Extensive content (2 points)	0 (0)	14 (7.0)	24 (12.1)	33 (16.6)	135 (67.8)	13 (6.5)

### Information Quality

Our results suggest that the general quality of the diabetes information videos on TikTok is acceptable. Overall, the videos published by the nonprofit organizations had the highest DISCERN scores, followed by those published by the health professionals and news agencies. The videos published by the for-profit organizations had the lowest total DISCERN scores, followed by those of the science communicators and general TikTok users. The mean numbers for the whole instrument indicate significant differences across the video sources, at α=.01 (Table 4).

Regarding reliability, videos published by the nonprofit organizations had the highest scores, while those from the for-profit organizations had the lowest scores. Our results suggest that nonprofit organizations, news agencies, and individual health professionals also contributed videos with relatively high reliability. The differences in reliability across the different video sources are significant, at α=.01.

In regard to treatment choices, diabetes-related videos on TikTok were of medium to low quality. Nonprofit organizations and health professionals contributed higher-quality videos on treatment choices than other sources; however, the differences are not significant.

**Table 4:** DISCERN scores of diabetes-related TikTok videos by source.

Video source	Reliability of the videos (items 1-8)^a^, mean (SD)	Quality of treatment choices (items 9-15)^b^, mean (SD)	Overall information quality (item 16)^c^, mean (SD)	Total DISCERN scores^d^, mean (SD)
Health professionals (n=138)	28.10 (3.59)	16.37 (5.00)	3.26 (0.67)	47.74 (7.71)
General users (n=12)	25.38 (3.19)	13.42 (3.63)	2.58 (0.51)	41.38 (6.14)
Science communicators (n=6)	25.00 (3.21)	15.25 (5.29)	3.08 (0.20)	43.33 (8.23)
News agencies (n=31)	28.48 (3.76)	16.02 (5.14)	3.23 (0.71)	47.73 (7.39)
Nonprofit organizations (n=7)	29.14 (2.25)	18.00 (3.40)	3.50 (0.50)	50.64 (4.61)
For-profit organizations (n=5)	24.20 (30.50)	13.20 (5.03)	2.60 (0.55)	40.00 (7.11)

^a^*P*=.005 (1-way analysis of variance).

^b^*P*=.23 (1-way analysis of variance).

^c^*P*=.004 (1-way analysis of variance).

^d^*P*=.009 (1-way analysis of variance).

## Discussion

### Principal Findings

This study systematically evaluated the information quality of diabetes-related videos on TikTok. According to a recent systematic review [13], the use of social media as a source of information is gaining in popularity among patients with diabetes. The various social media channels provide patients with a convenient means to seek medical knowledge and get social support [31]. While traditional social platforms (eg, Facebook, Twitter, and Instagram) have been widely investigated as channels of diabetes-related health communication [32], the role of emerging, mobile-based apps in disseminating diabetes knowledge is not yet fully understood. Our results reveal that TikTok is a powerful platform for disseminating diabetes-related information. The 199 diabetes videos examined in our study received 2.75 million “likes” and were commented on and shared thousands of times, which indicates that TikTok is a promising channel for health communication.

We identified 2 main categories of video uploaders (ie, individual and organizational users), each containing several more specific types of users. Individual users included health professionals, science communicators, general TikTok users; organizational users comprised news agencies, nonprofit organizations, and for-profit organizations. Health professionals contributed the most videos, while the for-profit organizations contributed the least. Many prior studies have suggested that health professionals and organizations can utilize social media for effective health communication and public health promotion [33,34]. Our study revealed that health professionals in China have been actively engaged in promoting diabetes knowledge via TikTok; however, nonprofit health organizations use this emerging video-based channel less frequently.

In terms of video content, the study found 3 types of imbalances. First, most of the videos were about type 2 diabetes mellitus, while very few videos discussed gestational diabetes mellitus and type 1 diabetes mellitus. Second, most of the videos were about disease management, but few fully addressed other aspects of content, such as the definition and symptoms of the disease, risk factors, evaluation, and outcomes. Third, when many videos are generally reliable, these videos were of average to fair quality concerning treatment choices. Prior studies suggest that patients with diabetes have various health information needs, including a need for information about treatment, course of the disease, abnormalities in glucose metabolism, progression of diabetes through their life cycle, coping techniques, and prevention [35,36]. Moreover, these information needs vary, depending on the type of diabetes mellitus. For example, young people with type 1 diabetes mellitus may be particularly interested in “diabetes through the life circle” [35]. Given the observed imbalances in video content, we suspect that current diabetes-related videos on TikTok cannot fully meet patients’ information needs. Therefore, we call for more pertinent videos to address patients’ comprehensive information needs.

Our study found that the quality of information in the videos differed with the type of source. Videos published by nonprofit organizations had the highest quality, while those from the for-profit organizations had the lowest quality. This finding is consistent with those of prior studies, which suggest that government-sponsored platforms are more likely to publish high-quality information than for-profit organizations [25]. Unfortunately, the videos contributed by the nonprofit organizations account for a mere portion of the total corpus of diabetes-related videos on TikTok. We suggest that government departments and public hospitals contribute more high-quality material and leverage the power of this social media channel to promote public health. Given the large variations in information quality from the different sources, we also suggest that patients exercise caution when using TikTok to obtain diabetes-related information.

### Limitations and Future Directions

The findings of this study should be viewed in light of several limitations. First, the study looked only at the quality of diabetes information, not the quality of communication. For example, we observed that the communication modalities varied largely in the TikTok videos. Some videos used rich materials (eg, illustrative images or cases and visual data) to communicate the information, while some were based on plain narratives. Unfortunately, the instruments employed in this paper targeted information quality and overlooked the quality of communication. We call for more empirical studies in the future to investigate the communication quality of diabetes-related videos on TikTok. Second, the videos included in our study were in Chinese; therefore, the findings cannot be applied to diabetes-related videos on TikTok in other languages (eg, English). We encourage future researchers to assess the information quality of diabetes-related videos in other languages to obtain deeper insight into quality issues with diabetes-related videos on TikTok. Third, there are many instruments for assessing the quality of health-related information, such as DISCERN, JAMA benchmarks, and the HONcode principles. This study employed the DISCERN instrument because it has proved effective for assessing the quality of videos on other platforms and apps (eg, YouTube). However, we encourage more studies using a variety of instruments to triangulate the validity of these findings in the future.

### Conclusions

This study assessed the information quality of 199 diabetes-related videos on the short video mobile app TikTok. The results show that the videos primarily addressed diabetes management but contained limited information on other types of content, such as the definition and symptoms of the disease, risk factors, evaluation, and outcomes. The overall quality of the diabetes videos was found to be acceptable on average, although it varied significantly, depending on the type of source. We conclude that the health information needs of patients with diabetes might not be fully met by watching TikTok videos, and patients should exercise caution when using TikTok for diabetes-related information.
